# Response to: Practical methods for incorporating summary time-to-event data into meta-analysis

**DOI:** 10.1186/1745-6215-14-391

**Published:** 2013-11-19

**Authors:** Yu Wang, Tingting Zeng

**Affiliations:** Laboratory of Molecular Diagnosis of Cancer, West China Hospital, Sichuan University, Chengdu, China; State Key Laboratory of Biotherapy, West China Hospital, Sichuan University, Chengdu, China; Adverse Drug Reaction Monitoring Centre of Chengdu, 366 Jincheng Avenue, High-tech District, Chengdu, China

## Abstract

A response to and comment on Practical methods for incorporating summary time-to-event data into meta-analysis, by Jayne F Tierney *et al*.

## Findings

As stated in the present article [1] by Tierney and colleagues, time-to-event outcomes were most appropriately analyzed using hazard ratios in meta-analyses. It provided step-by-step guidance on how to calculate hazard ratios and the associated statistics for individual trials, according to the information presented in the trial report. Among the 11 methods stated in this article, one of them presented suggested that, if it was reported in terms of *P*-value and events in each arm (and the randomization ratio was 1:1), these data can be used to estimate the O-E using Tierney’s equation 14:

In the example of the present article, the log-rank *P*-value of 0.075 gave a z-score of 1.78 according to the latter part of Tierney’s equation 14. Nevertheless, we found 2-sided *P*-value of 0.075 gave a z-score of 1.78 by using the net tools [2]. But the z-score for the 2-sided *P*-value should be divided by 2 as described by Tierney and colleagues. Thus we can speculate that the z-score for a *P*-value of 0.075 should be 0.89, which was produced from 1.78/2.

We also advertently found that a right-tailed *P*-value of 0.0375 gave a z-score of 1.78 by using the previous mentioned net tools [2], and 0.0375 by chance equals 0.075/2. So we speculated that the latter part of equation 14 required a more accurate representation of z-score and *P*-value as: z-score for (*P*-value/2).

Tierney and colleagues reported that if a 1-sided *P*-value was reported, it can be used directly to calculate the z-score without dividing by 2. According to Li [3], a 1-sided *P*-value was used in log-rank test or Cox regression, and the exact *P*-value was given in the table for Chi-square (right-tailed test). However, we verified that a right-tailed *P*-value 0.075 gave a z-score of 1.44 by using the previous mentioned net tools [2].

Therefore, we can conclude that a 2-sided *P*-value can be used to directly to obtain the z-score. The latter part of equation 14 need to be modified into: z-score for *P*-value. Otherwise, a 1-sided *P*-value divided by 2 is required to obtain the z-score and the latter part of equation 14 should be expressed more exactly as: z-score for (*P*-value/2).

## Authors’ reply

Jayne F Tierney, Lesley A Stewart, Davina Ghersi, Sarah Burdett and Matthew R Sydes.

We thank the correspondents for bringing to our attention the unfortunate ambiguity in our article [1]. It is correct that the *P*-value that should be divided by 2 in equation 14 and not the z-score. This was the intention, and is more explicit in the original equation [4], provided in the appendix of Tierney and colleagues [1]:

As suggested by the correspondents, equation 14 could be more precisely stated as:

and the related explanatory text altered accordingly: “As well as the events on each arm and overall, the z-score for half the two-sided *P*-value is required”

However, we disagree with the other points raised. It is suggested that one-sided *P*-values are used in log-rank tests and Cox regression models [3], whereas we find that it is standard practice to present two-sided (or two-tailed) *P*-values. Also, to our knowledge, all major statistical packages output two-sided *P*-values by default. It is therefore reasonable to assume that a *P*-value quoted in a trial publication will be two-sided unless otherwise stated, and to use this in equation 14. However, as described in the text [1]: “If a one-sided *P*-value is reported it can be used directly to obtain the z-score.”

This is justified by equation 14 being derived algebraically from the definition of the log-rank statistic as a normally-distributed random variable, and by the fact that a one-sided *P*-value (assuming the most extreme direction of effect) is half the magnitude of the corresponding two-sided *P*-value.

The correspondents go on to suggest that either a two-sided *P*-value or a one-sided *P*-value divided by 2 can be used directly in equation 14. In fact, this would produce an incorrect z-score and hazard ratio. Using the example in Tierney and colleagues, the z-score for the reported *P*-value of 0.075/2 (= 0.0375) is 1.78, as the correspondents themselves found using internet tools. This produces an O-E of 19.57 and hazard ratio of 0.85; the latter being identical to that reported in the trial publication [5]. If, as the correspondents suggested, we had used the reported *P*-value directly in equation 14, we would have obtained a z-score of 1.44, O-E of 15.82 and an incorrect hazard ratio of 0.88.

Finally, researchers wishing to estimate hazard ratios from published time-to-event data need not rely on deriving them manually using the equations provided [1], but instead can use the Excel spreadsheet that accompanies the paper. Moreover, they can use this to cross-check hazard ratios derived from different methods of estimation.
